# Supplementary material for: Learning to predict RNA sequence expressions from whole slide images with applications for search and classification
Source: Commun Biol. 2023 Mar 22;6:304. doi: 10.1038/s42003-023-04583-x (PMC10033650; doi:10.1038/s42003-023-04583-x)
Supplement: Supplementary file 3 — Supplementary Information [file 42003_2023_4583_MOESM3_ESM.pdf]

Supplementary Information

Supplementary Table 1: TCGA kidney dataset split for transcriptomic learning (the number of cases, slides, and FPKM files per subtype per subset).

	Train			Validation			Test		
Subtype	Cases	Slides	FPKMs	Cases	Slides	FPKMs	Cases	Slides	FPKMs
ccRCC	369	372	373	43	43	45	46	47	48
crRCC	47	47	47	8	8	8	7	7	7
pRCC	195	215	195	26	28	26	24	26	24

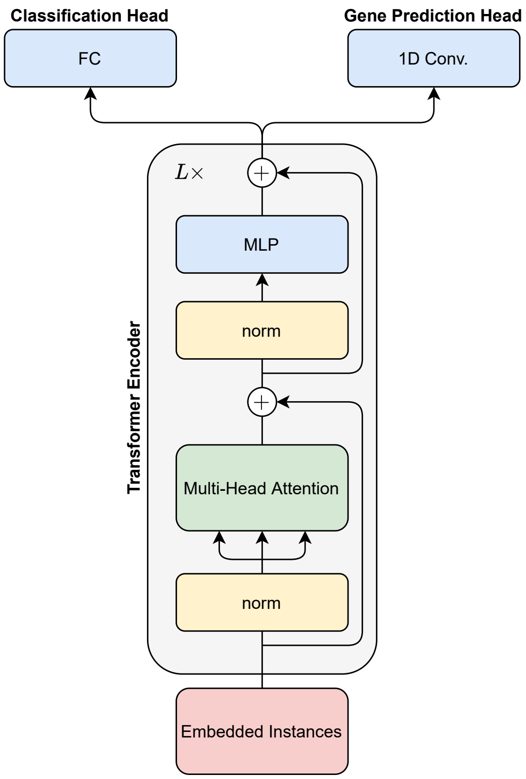

(a) **tRNAsformer** model

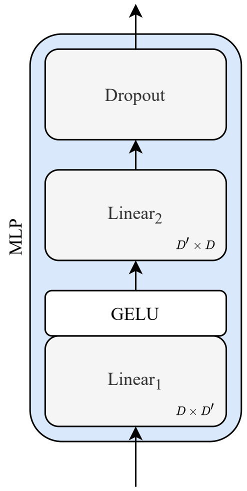

(b) structure of the *MLP* block

Supplementary Figure 1: The **tRNAsformer** model architecture – (a) a standard Transformer Encoder comprises layernorm, multi-head attention, multi-layer perceptron block, and residual skip connections. Because it is a multi-head self-attention module, the first layernorm's output embedding is provided to the multi-head attention as the query, key, and value. Each model can have L blocks of Transformer Encoder. The classification head transforms the internal representation to the number of classes, whereas the gene prediction head maps it to the number of genes. (b) a detailed diagram of multi-layer perceptron block (*MLP*). The letter D refers to the size of internal representation in the Transformer Encoder, and $\frac{D'}{D}$ is referred to as *MLP* ratio.

Supplementary Table 2: The number of parameters and one epoch's wall clock processing time for **tRNAsformer** and $\text{HE2RNA}_{\text{bb}}$ models. When the minibatch is set to 64, the processing time is the wall clock time for one epoch of training or validation.

		Processing time (s)	
Model	Number of parameters	Training	Validation
$\text{tRNAsformer}_{L=1}$	14,429,876	128	61
$\text{tRNAsformer}_{L=2}$	16,204,340	133	60
$\text{tRNAsformer}_{L=4}$	19,753,268	146	61
$\text{tRNAsformer}_{L=8}$	26,851,124	173	64
$\text{tRNAsformer}_{L=12}$	33,948,980	205	65
$\text{HE2RNA}_{\text{bb1024}}$	34,687,025	335	81

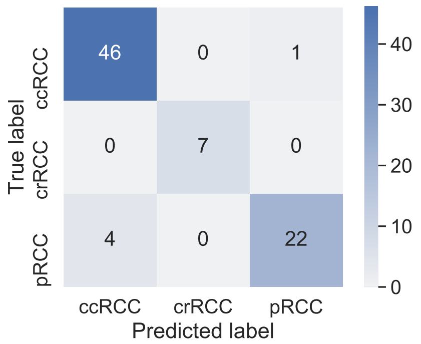

(a) $\text{tRNAsformer}_{L=1}$

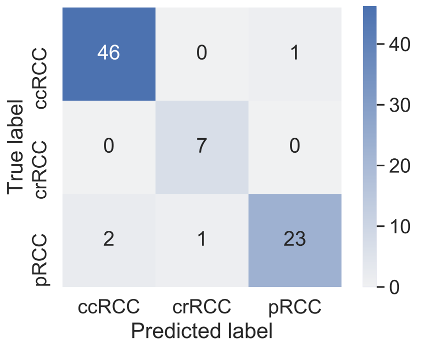

(b) $\text{tRNAsformer}_{L=2}$

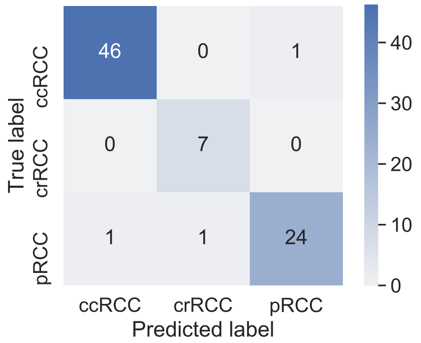

(c) $\text{tRNAsformer}_{L=4}$

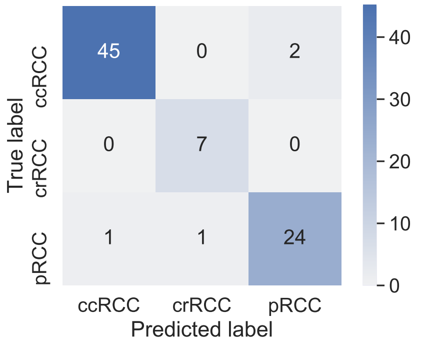

(d) $\text{tRNAsformer}_{L=8}$

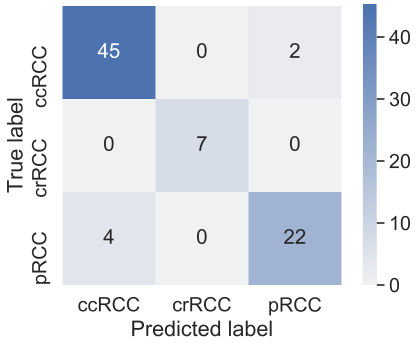

(e) $\text{tRNAsformer}_{L=12}$

Supplementary Figure 2: The confusion matrices for different models applied on 8,000 bags created from 80 TCGA test WSIs. (a)-(f) are for tRNAsformer_L, $L = (1, 2, 4, 8, 12)$, respectively.

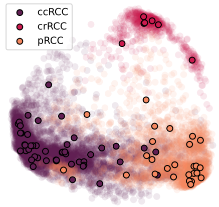

(a) tRNAsformer$_{L=1}$

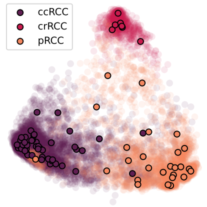

(b) tRNAsformer$_{L=2}$

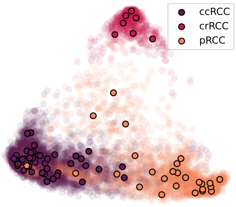

(c) tRNAsformer$_{L=4}$

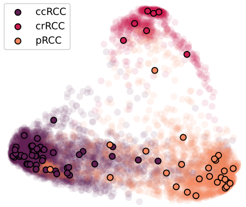

(d) tRNAsformer$_{L=8}$

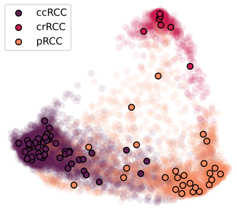

(e) tRNAsformer$_{L=12}$

Supplementary Figure 3: The two-dimensional PCA projection of TCGA test WSI features. (a)-(f) are for tRNAsformer$_L$, $L = (1, 2, 4, 8, 12)$, respectively. Each TCGA test WSI is represented by 100 bags of features. All bags of features associated with the test set are shown with transparent circles. The average of PCA projection of each WSI (average of 100 bags associated with each WSI) is shown in bold circles with black edges.

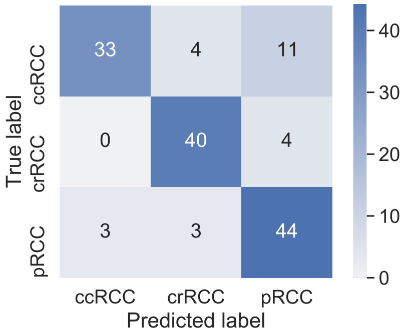

(a) tRNAsformer$_{L=1}$

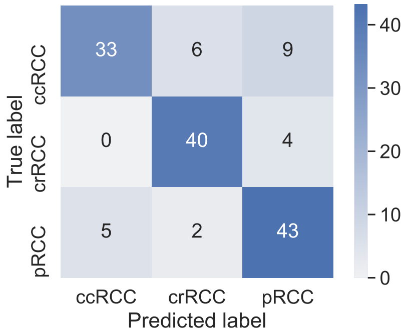

(b) tRNAsformer$_{L=2}$

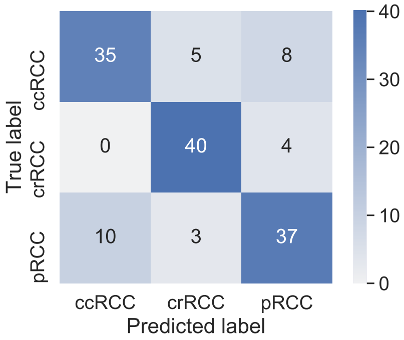

(c) tRNAsformer$_{L=4}$

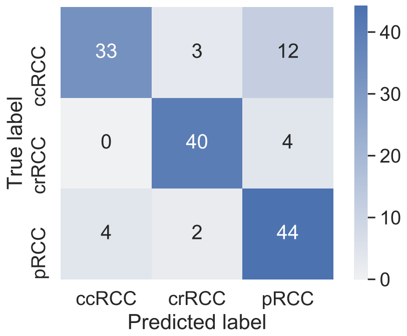

(d) tRNAsformer$_{L=8}$

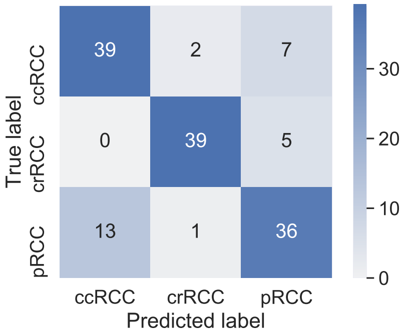

(e) tRNAsformer$_{L=12}$

Supplementary Figure 4: The confusion matrices for different models applied on 14,200 bags created from the external dataset WSIs. (a)-(d) are for tRNAsformer$_L$, $L = (1, 2, 4, 8, 12)$, respectively.

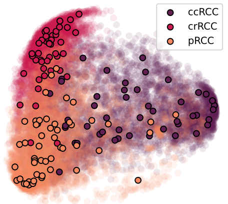

(a) tRNAsformer$_{L=1}$

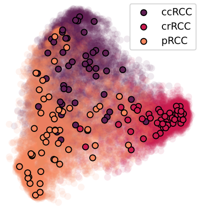

(b) tRNAsformer$_{L=2}$

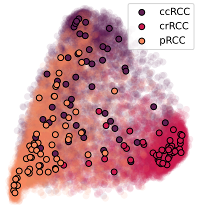

(c) tRNAsformer$_{L=4}$

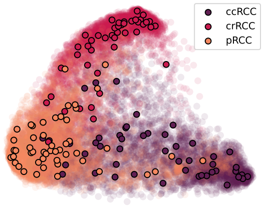

(d) tRNAsformer$_{L=8}$

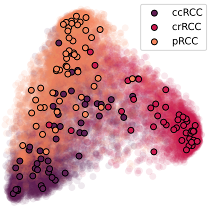

(e) tRNAsformer$_{L=12}$

Supplementary Figure 5: The two-dimensional PCA projection of the external dataset WSI features. (a)-(f) are for tRNAsformer$_L$, $L = (1, 2, 4, 8, 12)$, respectively. Each external test WSI is represented by 100 bags of features. All bags of features associated with the test set are shown with transparent circles. The average of PCA projection of each WSI (average of 100 bags associated with each WSI) is shown in bold circles with black edges.
